# Sperm competition-induced plasticity in the speed of spermatogenesis

**DOI:** 10.1186/s12862-016-0629-9

**Published:** 2016-03-08

**Authors:** Athina Giannakara, Lukas Schärer, Steven A. Ramm

**Affiliations:** Evolutionary Biology, Bielefeld University, Morgenbreede 45, 33615 Bielefeld, Germany; Evolutionary Biology, Zoological Institute, University of Basel, Vesalgasse 1, 4051 Basel, Switzerland

**Keywords:** phenotypic plasticity, sexual selection, spermatogenesis, sperm competition, testicular function

## Abstract

**Background:**

Sperm competition between rival ejaculates over the fertilization of ova typically selects for the production of large numbers of sperm. An obvious way to increase sperm production is to increase testis size, and most empirical work has focussed on this parameter. Adaptive plasticity in sperm production rate could also arise due to variation in the speed with which each spermatozoon is produced, but whether animals can respond to relevant environmental conditions by modulating the kinetics of spermatogenesis in this way has not been experimentally investigated.

**Results:**

Here we demonstrate that the simultaneously hermaphroditic flatworm *Macrostomum lignano* exhibits substantial plasticity in the speed of spermatogenesis, depending on the social context: worms raised under higher levels of sperm competition produce sperm faster.

**Conclusions:**

Our findings overturn the prevailing view that the speed of spermatogenesis is a static property of a genotype, and demonstrate the profound impact that social environmental conditions can exert upon a key developmental process. We thus identify, to our knowledge, a novel mechanism through which sperm production rate is maximised under sperm competition.

**Electronic supplementary material:**

The online version of this article (doi:10.1186/s12862-016-0629-9) contains supplementary material, which is available to authorized users.

## Background

Whilst only a single sperm is required to fertilize each ovum, the number of sperm produced by males (or in the case of simultaneous hermaphrodites, the male sex function) usually greatly exceeds the number of ova produced by females (or the female sex function). This apparent profligacy likely evolved as a direct consequence of disruptive selection on gamete size and number during the evolution of anisogamy, and is maintained because of sperm competition, which occurs when the ejaculates from two or more sperm donors compete over fertilization and which creates an evolutionary arms race between conspecific rivals over sperm production [1–4].

Evidence that sperm competition drives patterns of sperm production is widespread, typically involving measures of testis size [5, 6]. For example, the importance of sperm competition in driving sperm production capacity has been inferred from comparisons of the relative testis sizes of different species differing in sperm competition level across a wide range of animal taxa (e.g. [5, 7, 8]), from the experimental evolution of altered testis size under elevated or diminished levels of sperm competition (e.g. [9, 10]) and from the systematic differences in relative testis size observed in individuals adopting alternative male reproductive tactics differing in their expected incidence of sperm competition (e.g. [11–13]).

Despite increased testis size undoubtedly reflecting adaptation to heightened sperm competition, testis size cannot be directly equated with sperm production rate. This is because i) testis size is a static rather than a dynamic measure of sperm production (i.e. although testis size might vary over time, any one “snapshot” simply measures the size of the factory, but not the activity within it or its output [6, 14]); and ii) doing so would ignore many other potentially significant sources of variation in sperm production rate within the testis (reviewed in [15, 16]). These include the histological and logical organisation of spermatogenesis as well as its kinetics, and accumulating evidence suggests that these sources of variation indeed warrant further investigation from a sperm competition perspective [17–21]. In particular, recent experimental studies indicate that individuals of several taxa can upregulate sperm production in response to environmental cues of heightened sperm competition level, and that this can occur, at least in part, independently of testis size [22–25] (see also [26, 27] for further recent examples of sperm production plasticity in which the relation to testis size was not investigated). Such a disproportionate increase in sperm production under social or ecological conditions leading to heightened sperm competition implies that there must be intraspecific plasticity in additional spermatogenic parameters, such as for example the proportion of spermatogenic tissue found within the testis or in the kinetics of spermatogenesis itself. However, despite the well-reported interspecific variation in these parameters driven by sperm competition (e.g. [18–21, 28]), it has not been investigated whether or not the kinetics of spermatogenesis can be plastically adjusted within species according to the prevailing sperm competition conditions. That was therefore the aim of this study.

The simultaneously hermaphroditic marine flatworm *Macrostomum lignano* has recently emerged as a useful model organism in various fields of biology [29, 30], in part due to its short generation time, transparency and ease of experimental manipulation. Notably in the present context, *M. lignano* has been the subject of intensive investigation from the point of view of sex allocation theory [31]. These studies have revealed that *M. lignano* flatworms are able to dynamically adjust resources allocated to sperm (and ovum) production in response to changes in social group size [6, 23, 32–34], a parameter which accurately reflects mating group size (i.e. the average number of mating partners plus one) and thus sperm competition level in this species [34, 35]. Importantly, however, the number of sperm produced – as measured by the filling rate of the seminal vesicle upon a worm’s social isolation – increases disproportionately with increases in testis size [23]. This is unlikely to be due to changes in the proportion of spermatogenic tissue, because the testis of *M. lignano* contains very few non-spermatogenic cells [15, 23] and the number of both proliferating cells [6] and differentiating spermatids [36] increases linearly with testis size. Sperm morphology is relatively stable across different social environments [33, 37]. These previous findings – together with the fact that a synthetic analog of thymidine, bromodeoxyuridine (BrdU), can be readily administered to label and visualize proliferating cells within the testis of this species [6, 38] – make *M. lignano* a highly attractive model to test the resulting hypothesis that one way in which sperm competition drives an increased sperm production rate is through altering the kinetics of spermatogenesis [23]. Applying these techniques, we here establish for the first time, to our knowledge, that there is indeed social environmentally-induced plasticity in the speed of spermatogenesis: worms raised under higher levels of sperm competition produce sperm faster.

## Results

### Testis size plasticity

**Fig. 1:**
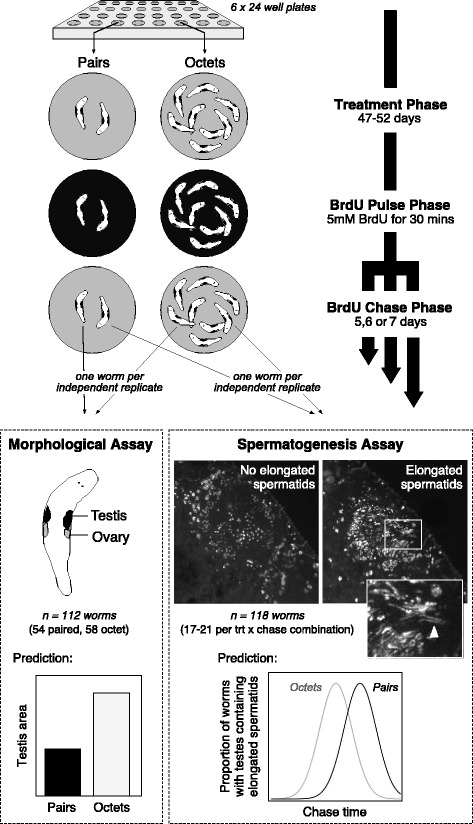
Experimental design and predictions of the test for plasticity in testis size and spermatogenesis kinetics according to the social environment in *Macrostomum lignano*. The experiment comprised a treatment phase in which flatworms were allocated to and maintained in standardized group sizes of either two or eight worms (‘pairs’ and ‘octets’, respectively). They were then pulsed for 30 min with 5 mM BrdU in order to label a pool of S-phase cells, which was then visualized after a variable chase phase of 5, 6 or 7d. One experimental subject per replicate was then used for a morphological assay of testis area and a second for a spermatogenesis assay that meant scoring their testes for the presence of elongated BrdU-labelled spermatids. The two images are z-projections of the testis region of *M. lignano* derived from confocal laser scanning micrographs of worms fixed 4 and 5 days following BrdU pulse (*left* and *right*, respectively), to illustrate the morphological appearance of testes containing no BrdU-labelled elongating spermatids and testes containing BrdU-labelled elongating spermatids, respectively (images modified from Schärer *et al.* 2007). Anterior is to the top left. The inset on the right-hand image highlights an area towards the centre of the testis where elongating spermatids are clearly visible (indicated by the arrowhead). For full details of the experimental design in this study, see Methods. Specific predictions depicted in the bottom panels are explained in the Results

We performed an experiment to manipulate the levels of sperm competition by keeping worms (*n* = 720) in two different social group sizes (‘pairs’ and ‘octets’), followed by labelling and immunocytochemical tracking of proliferating testicular germ cells, to a) confirm the expected phenotypic plasticity in testis size usually observed in these worms in response to altered social and mating group size (e.g. [32, 34]), and b) test the main hypothesis of this study that worms kept in larger social groups also increase the speed of spermatogenesis (an overview of the experiment is shown in Fig 1; full details are provided in the Methods section).

**Fig. 2:**
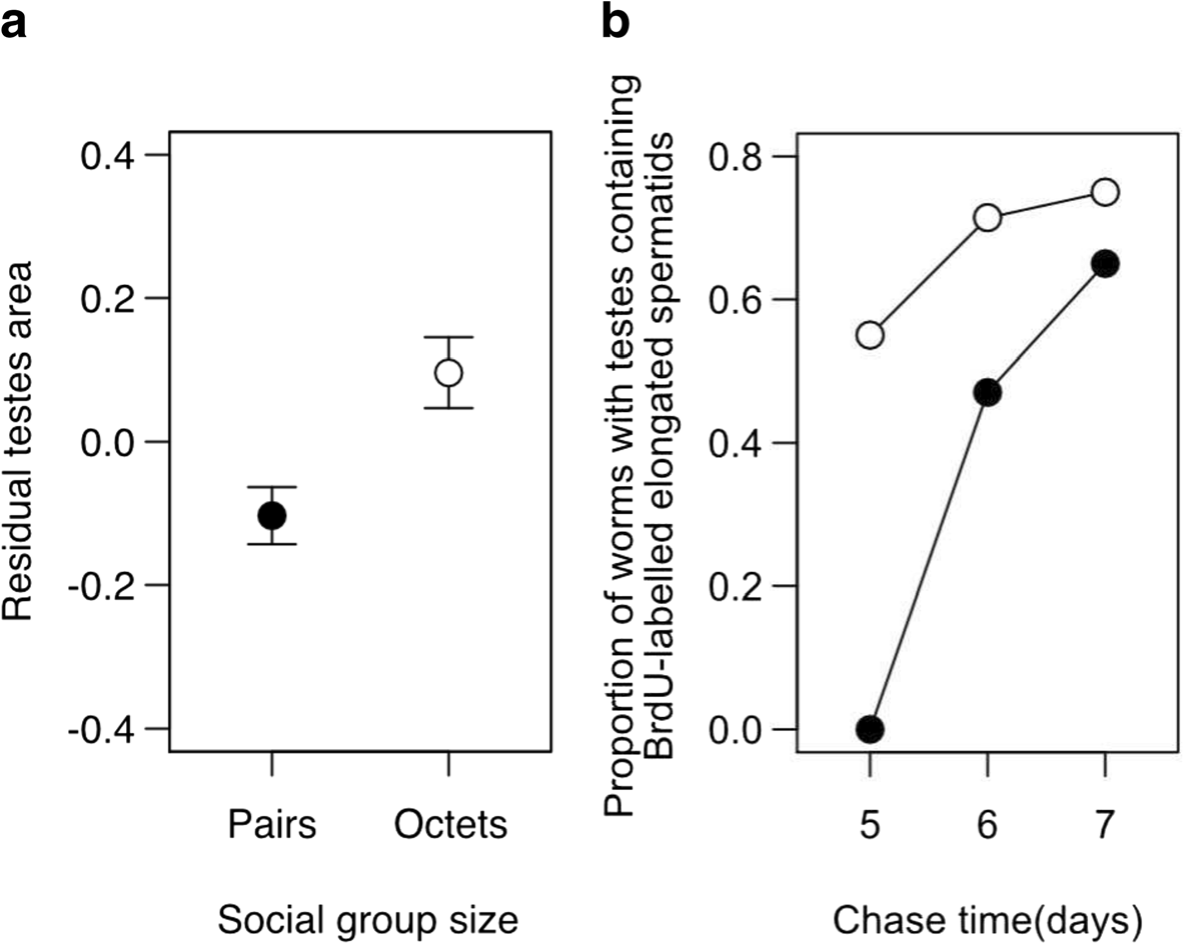
Plasticity in testis size and spermatogenesis kinetics according to the social environment in *Macrostomum lignano*. In octets compared to pairs, flatworms exhibit **a** greater relative investment in testes (i.e., a larger residual testis area) and **b** an increased speed of spermatogenesis, here visualized as the observed proportion of worms exhibiting BrdU-pulse labelled elongated spermatids in their testes, for octets (open circles) and pairs (filled circles) 5-7d after a 30 min pulse administration of BrdU

As expected, worms raised in octets exhibited significantly larger testes than worms raised in pairs, both in absolute terms (mean ± SE testis area, pairs: 12,485 ± 844 μm^2^, octets: 17,993 ± 989 μm^2^; *t*_110_ = 4.29, *P* < 0.0001), and according to our measure of relative investment accounting for body size differences between worms (mean ± SE residual testis area: pairs: −0.103 ± 0.040, octets: 0.096 ± 0.049; *t*_110_ = 3.13, *P* = 0.002, Fig 2a) Note that an analysis of covariance (ANCOVA) on relative testis area including body area as a covariate produced qualitatively identical results (not shown) and that there was no difference between pairs and octets in residual ovary size (*t*_110_ = 0.06, *P* = 0.9). The testis size response to different sperm competition environments is consistent with several earlier studies in this species, and with both sperm competition and sex allocation theory [39, 40].

### Plasticity in spermatogenesis kinetics

To test the specific hypothesis that this testis size response is accompanied by changes in the speed with which sperm are produced within the testis, i.e. that the higher levels of sperm competition experienced in octets compared to pairs induces an increased speed of spermatogenesis, we next performed a BrdU pulse-chase assay to track the progress of a pool of BrdU-labelled spermatogenic cells and thereby measure the speed of spermatogenesis (see Methods). Briefly, administration of a short pulse of BrdU results in its incorporation into any cells in S-phase, which in the *M. lignano* testis means either spermatogonial stem cells about to divide or primary spermatocytes replicating their DNA before they enter meiosis (there is no mitotic expansion in this species [38]), resulting in a wave of BrdU-labelled cells passing through the testis. This can be used to assess potential differences in spermatogenesis kinetics because for each worm the appearance (or not) of BrdU-labelled elongated spermatids provides a clear morphological checkpoint, indicating that a late stage in spermatogenesis has been reached (or not). Using this approach, we can derive two clear predictions for the chase time window we investigated following BrdU administration. First, if elongated spermatids are produced sooner in octets compared to pairs, at the beginning of the observation window (5 days after BrdU pulse) we should see labelled elongated spermatids in the testes of a greater proportion of octets compared to pairs. Second, if at this time the elongation wave is closer to its peak in octets than in pairs, the probability of observing worms with testes containing elongated spermatids should increase more strongly over the five-to-seven-day chase time window in pairs than in octets. Both predictions are strongly supported by our data (Fig 2b).

On chase day 5, over half of the octets exhibited BrdU-labelled elongated spermatids (11/20 = 55 %), whereas none of the pairs (0/20) did so, indicating that the distribution of elongated spermatids is highly skewed with respect to social group size treatment (Pearson χ^2^ = 15.17, d.f. =1, *P* < 0.0001).

Over the course of the experiment, a generalised linear model analysis (see Methods) revealed that there was indeed a significant chase time × group size interaction, with the probability of observing elongated spermatids increasing more strongly for worms from pairs than from octets (Table 1).

**Table 1:** Generalized linear model of the presence of elongated spermatids in the testes of *M. lignano* flatworms from a BrdU pulse-chase experiment. Following the administration of a 30 min BrdU pulse on Day 0, worms were maintained in two different social environments (pairs, octets) until being fixed for immunocytochemistry on Days 5, 6 and 7. The model treats the presence of BrdU-labelled elongated spermatids in their testes as the binomial response variable with three explanatory terms: group size, chase time, and a group size × chase time interaction. Total *n* = 118

Term	Estimate	S.E.	χ^2^	d.f.	*P*
(Intercept)	−2.03	2.06			
Chase time	0.46	0.35	16.66	1	<0.0001
Group size	−9.38	3.63	12.76	1	0.0004
Chase time × Group size	1.30	0.59	5.40	1	0.02

These two patterns can clearly be observed in Fig. 2b, with a far greater proportion of octets exhibiting elongated spermatids in their testes on chase day 5, and a more strongly increasing proportion of pairs with elongated spermatids over time. This fits with our underlying hypothesis that the wave of observed elongated spermatids appearing in the testis following a single BrdU pulse is essentially similar for octets and pairs, except for the crucial difference that it occurs sooner in octets owing to their increased overall speed of spermatogenesis.

## Discussion

The central importance of spermatogenesis to male reproductive success is obvious, and this has long been recognised in the evolutionary literature. However, it is usually assumed that – although differing widely between species – the duration of spermatogenesis is a relatively fixed parameter within species, under the assumption that sperm of a certain species-specific morphology take a certain amount of time to produce (reviews in [41, 42]). There is some previous evidence that different genotypes of the same species exhibit differences in spermatogenesis kinetics [14], but our results clearly point to an even more dynamic scenario. The environmentally-induced increase in the speed of spermatogenesis we have documented likely contributes to the higher rate of sperm production observed under higher levels of sperm competition in *M. lignano*, as recently proposed by Schärer & Vizoso [23]. We therefore interpret this finding as an adaptive response to maximize sperm production rate, under conditions where sperm competitive ability becomes increasingly important to male reproductive success.

As far as we are aware, this represents the first report of intra-specific variation in spermatogenesis kinetics being driven by sperm competition. Although the evolutionary rationale for such a response seems clear – conforming to both sperm competition [40] and sex allocation theory [39] – most research to date in this area has focussed strongly on more easily measured parameters influencing sperm production rate, such as different measures of relative testis size (reviewed in [6, 15]). Given that *M. lignano* also increases its testis size in larger social groups, our study implies that sperm production rate is maximized by a combination of these two responses, i.e. by increasing both the amount of spermatogenic tissue and the efficiency (per unit volume) with which this tissue produces sperm. Extrapolating from our data, we would estimate that the total shift in the duration of spermatogenesis induced by living in octets rather than pairs was around 1.5 days. Such an effect size could fully account for the disproportionate increase in sperm production rate (as measured by seminal vesicle filling rate) that could not be explained by testis size differences between pairs and octets in the earlier study of Schärer and Vizoso [23]. We calculate that these effects are likely to be of approximately equal magnitude, with just over half (55.5 %) of the 37 % difference in sperm production rate between pairs and octets in that study attributable to testis size differences between treatments [19], and the remainder (44.5 %) due to a testis size-independent source of variation. Our results would now appear to identify that source, and thus strongly imply that differences in sperm production due to plasticity in the speed of spermatogenesis can be substantial, at least in this species. Certainly our results add to accumulating evidence that testis size alone – although undoubtedly important and often a useful proxy – is far from a complete measure of male adaptation to engage in sperm competition through increasing sperm production [15, 16, 23].

The proximate cues driving plasticity in sex allocation and spermatogenesis kinetics are currently unclear. Previous work in *M. lignano* has clearly established that social group size affects mating group size and thus sperm competition level [34], driving plastic shifts in sex allocation (e.g. [32, 34]), mating rate [43] and a correlated difference between “pairs” and “octets” in the size of the seminal vesicle (see e.g. [23, 32]). The latter is usually found to be smaller in octets, which presumably reflects the fact that worms in octets are using sperm as soon as they are produced, thus preventing their accumulation in the seminal vesicle. Cues such as mating rate or the rate of emptying and re-filling of sperm reserves in the seminal vesicle might function to signal to the testis to regulate sperm production, and are potential proximate cues underlying the response we have described here. They should generally be highly predictive of levels of sperm competition experienced by these worms. In addition to the precise cues involved, we also do not currently know how the subsequent responses in terms of both the machinery and speed of spermatogenesis are achieved. We have, however, recently identified many testis-specific candidate genes [44] that are differentially expressed in different social group sizes in this species [45], opening up the possibility of manipulating specific spermatogenesis candidates by RNA interference to characterize their functions (e.g. [46, 47]) and conceivably to being able to manipulate plasticity and thereby assess its fitness consequences.

The only previous reports we are aware of for any environmentally-induced intra-specific variation in spermatogenesis kinetics are linked to temperature. Studies in fish, reptiles and mammals have shown that – within a certain tolerable range – spermatogenesis kinetics are affected by temperature (e.g. [48–51]). Temperature likely also plays a role in explaining the faster first round of spermatogenesis observed in young male mice, which occurs before the testes are fully descended [52]. All of these temperature effects, however, appear likely to reflect a rather generalized direct influence of temperature on all biological processes within the organism in question, not just the testis. We would therefore argue that the effect on spermatogenesis we have identified here is a distinct and novel phenomenon, representing a likely adaptive shift in spermatogenesis speed induced by the increased levels of sperm competition the worms encountered in our experiment.

A significant open question concerns the taxonomic generality of our findings. Given the relative difficulty of estimating spermatogenesis kinetics and other dynamic measures of sperm production, it is perhaps unsurprising that most evolutionary studies to date have relied on static measures of sperm production, such as relative testis size, or other likely proxies of sperm production such as sperm numbers in storage or in ejaculates. But there seems little reason to expect *a priori* that similar responses might not be selectively favoured also in other animal groups. Certainly there is evidence in other groups that overall sperm production rate exhibits plasticity in response to changes in the sperm competition levels [22, 24–27]. Nevertheless – and despite some highly conserved aspects of spermatogenesis [53] – the huge differences between taxa in testicular architecture and final sperm morphology likely place very different demands on the machinery of spermatogenesis [15], and this may well differentially affect the scope for plasticity in different spermatogenic parameters.

Another important consideration here will be identifying the nature and extent of the costs (or trade-offs) of producing sperm at a faster rate, because in the absence of such costs it is difficult to see why a testis developing in a low sperm competition environment should be built for sub-optimal performance. One possible explanation is that it is important to retain some flexibility in sperm production rate, and one potentially quite efficient way of achieving that is by varying the workload of a testis of a given size so that it is producing sperm at a faster or a slower rate (altering testis size is of course another means to the same end, though this response may take longer to implement). Notably, however, it seems that testes with differing workloads in *M. lignano* do not produce sperm with markedly different morphologies [33, 37].

Finally, environmental variation in the sperm competition level on a temporal scale permitting adaptive responses in testicular function is of course a prerequisite for selection for plasticity in spermatogenesis; if sperm competition levels are either stable or change very rapidly and unpredictably, then there may be little reason to expect phenotypic plasticity in spermatogenesis kinetics or any other sperm production trait to evolve. In *M. lignano*, we know that keeping worms in social group sizes of two or eight worms likely represents a large part of the range of mating group size observed in this species [34], and that plastic responses in testis size to altered group size can occur even over much more rapid timescales than those investigated here [54]. Thus we expect that the stable social group size we employed for our experimental design was not crucial to the results, and that worms can quickly adjust spermatogenic parameters in response to changes in the social environmental conditions. Exactly how variable social conditions are in nature is currently unclear, but field-collected worms are found at a wide range of densities (LS, unpubl. observations), implying that there is likely to be substantial variation in mating group size also in the wild.

## Conclusions

In conclusion, by demonstrating plasticity in the speed of spermatogenesis we have identified a novel mechanism through which sperm production rate is maximised under sperm competition. Our findings have broad implications for our understanding of the key biological process of spermatogenesis, and highlight the profound influences that environmental conditions can exert on fertility.

## Methods

### Study animal

*Macrostomum lignano* (Macrostomorpha, Platyhelminthes) is an outcrossing, free-living marine flatworm and a member of the interstitial sand fauna of the Northern Adriatic Sea [29, 32]. In the laboratory it is kept in Petri dishes containing 32‰ artificial sea water (ASW) or f/2 medium [55] and fed *ad libitum* on *Nitzschia curvilineata* diatoms, held at constant temperature and relative humidity (~20 °C and ~60 % respectively) and on a 14:10 h light:dark cycle. Under these standard culturing conditions, adult worms from outbred lines (body length ca. 1.5 mm) lay ca. 1–2 eggs per day, with eggs hatching after ca. 5 days and individuals reaching sexual maturity ca. 2 weeks later. In this study we used an inbred line, DV1, which was created by 24 generations of full- and half- sib inbreeding, as described in [34], and has a somewhat longer generation time of approximately 4 weeks. The DV1 culture used has been maintained at Bielefeld University since 2012, when it was obtained from the original DV1 culture generated and maintained at the University of Basel (see ref. [32]). No additional fieldwork or ethical permissions were required for the experiments reported here. A major methodological advantage of *M. lignano* is its near transparency, permitting *in vivo* measurement of relevant reproductive organs [32], as well as the availability of BrdU-labelling methods to track stem cell proliferation, spermatogenic investment, and the process of spermatogenesis [6, 38, 56].

### Group size treatment phase

To produce similarly-aged hatchlings to be assigned as experimental subjects, 150 adult worms were taken from a mass DV1 culture and placed in a Petri dish containing a dense layer of algae to lay eggs. Beginning one week later, hatchlings produced by these worms were collected every day and randomly assigned to social groups of either two (‘pairs’) or eight (‘octets’) worms. All groups were housed in ca. 1 ml ASW in wells of 24-well plates (TPP, Trasadingen, Switzerland), with each plate containing 12 pairs and 12 octets in a balanced arrangement, for a total of 6 plates (i.e. 720 worms in total). All worms within the same group and all worms on the same plate were always allocated on the same day and thus had the same age. Worms were fed *ad libitum* with *N. curvilineata* throughout the experiment, with octet wells receiving four times as much algae as those of pairs. 13–15 days later, worms were transferred for the first time into new 24-well plates under the same conditions and thereafter transferred to new plates every 6–7 days, in order to avoid the accumulation of offspring of these focal worms that would alter the social group size composition (given that eggs take 5 days to hatch, by the time the adult worms were transferred any hatchlings could be 2 days old at most). As normally occurs in such group size experiments [34, 43], several worms were lost during the transferring procedures, resulting in a reduction in group size. Any replicates with missing worms were excluded from further processing. In total, at the end of the group size experimental phase, *n* = 124 remaining worms had been assigned into 63 pairs and *n* = 488 worms into 61 octets.

### BrdU pulse-chase phase

At 47–52 days of age, all experimental worms were pulsed with BrdU (in three batches, such that two consecutive plates were always processed on the same day). All replicates in each plate were retained in their original group composition, and incubated in a 1:10 mixture of 50 mM BrdU (5-bromo-2ʹ-deoxyuridine, B5002, Sigma-Aldrich Biochemie GmbH, Hamburg, Germany) and ASW (i.e., final BrdU concentration: 5 mM) in separate wells of a 96-well PCR loading plate (Eppendorf, Germany) for 30 minutes in the dark. After washing three times in ASW to remove excess BrdU, worms were returned, still in their original group constitution, to the standard group size conditions in 24-well plates, where they remained for a further 5, 6 or 7 days depending on the randomly allocated 'chase time' to which they had been assigned, with all six 'group size' × 'chase time' combinations approximately equally represented on each plate (2–4 replicates per plate).

At the end of the chase phase of the experiment, when worms were 52–59 days old, one worm from each replicate was randomly selected for morphological assessment of testis size (see “Morphometry assay” below) and a second randomly selected for visualization of BrdU-labelled cells and assessment of its spermatogenic stage (see “Spermatogenesis assay” below).

### Morphometry assay

Testis size was estimated using standard techniques for this species [32] (Fig. 1). Briefly, one randomly selected worm from each replicate was (within 2 hours after its selection) placed on a microscope slide in a drop of 1:1 mixture of MgCl_2_ and ASW (total volume 40 μl) for approximately 4 minutes, until it was anaesthetized. It was then squeezed dorsoventrally with a cover slip using two small squares of plastic film of standard thickness as spacers [32]. Squeezed worms were observed under 100-400x magnification using an Olympus BX50 microscope (Olympus Deutschland GmbH, Hamburg, Germany) coupled to a Canon EOS 600D camera connected to a computer using the Zoom Browser EX version 6.9.0a software, permitting digital photos of relevant morphological features to be captured. Photos were then processed using ImageJ (http://imagej.nih.gov/ij/) to calculate whole body area and the area of both testes. During the image acquisition and measuring steps, the observer was blind with respect to the treatment group of each worm. One pair and one octet worm was crushed during the squeezing procedure and both were excluded from the analysis, as were 5 worms from the paired treatment that appeared to be malformed (poor development, lack of seminal vesicle and/or stylet, hypertrophic testis, multiple stylets), as well as 2 pair worms and 2 octet worms for which it was not possible to obtain a complete set of photos. This resulted in a final dataset for the Morphometry assay of *n* = 54 paired and *n* = 58 octet worms.

### Spermatogenesis assay

The other randomly selected worm from each replicate was processed so as to visualize BrdU-labelled cells in the testes and thereby score their spermatogenic stage (i.e. how far the label has advanced), based on previous protocols [6, 38]. For logistical reasons, the 2–4 worms coming from independent replicates belonging to the same group size × chase time combination on each plate were processed together solely for the visualization step (but then mounted on separate slides – see below). At the appropriate chase day (5, 6 or 7 days after BrdU pulse), worms were anaesthetized in a mixture of MgCl_2_ and ASW for 15–20 minutes, slowly increasing the concentration to 1:1, and then fixed for 60 minutes in 4 % paraformaldehyde (PFA) in phosphate-buffered saline (PBS). The fixed worms were then washed three times in PBS-T (i.e. PBS plus 0.1 % Triton X-100) for 15 minutes, and then soaked in PBS-T for a further 60 minutes. Next, they were permeated with 0.15 mg/ml Protease XIV (erroneously reported as 0.15 μg/ml in some earlier studies) and PBS-T at room temperature. The activity of the protease was visually checked and stopped after approximately 30 minutes with cold 0.1 N HCl. The worms were then transferred in 2 N HCl for 60 minutes to denature DNA, washed with PBS-T and then blocked for 30 minutes with BSA-T (i.e. PBS-T plus 1 % bovine serum albumin). The worms were then incubated overnight in a 1:400 mixture of the primary rat anti-BrdU antibody (ab6326, Abcam Limited, Cambridge, UK) and BSA-T at 4 °C. On the following day, they were washed three times with PBS-T for 15 minutes and transferred into a 1:200 mixture of the fluorescein isothiocyanate (FITC)-conjugated secondary antibody (goat F (ab’)2 anti-rat IgG, ab6115, Abcam Limited, Cambridge, UK) in BSA-T for 60 minutes in the dark. Following a triple washing step with PBS-T for 15 minutes, worms were placed briefly in PBS for 1 minute and then mounted individually on a microscope slide (i.e., one worm per slide) in 22 μl of Vectashield (Vector Laboratories, Inc., Burlingame, CA, USA), covered with a cover slip and sealed. Mounted worms were given a unique ID and stored at −20 °C until all replicates had been processed and the spermatogenic stage of each worm could be assessed in a single session. One pair replicate was lost during the visualization procedure, meaning that in total *n* = 62 worms kept in pairs and *n* = 61 worms kept in octets were successfully processed, comprising *n*_5,P_ = 21 pairs and *n*_5,O_ = 20 octets fixed after a 5d chase period; *n*_6,P_ = 20 pairs and *n*_6,O_ = 21 octets fixed after 6d; and *n*_7,P_ = 21 pairs and *n*_7,O_ = 20 octets after 7d.

To score the spermatogenic stage, two researchers blind to the treatment group and chase day of each worm independently viewed the mounted worms under epifluorescence at 400x magnification using a Nikon Ni-U microscope (Nikon GmbH, Düsseldorf, Germany) and scored the elongation status of cells within the testis (0 = no BrdU-labelled elongated spermatids observed, 1 = BrdU-labelled elongated spermatids observed in at least one testis) (Fig. 1). Using such a binary response potentially creates a bias in our observations, because the fact that we strongly expect that worms in octets have higher testicular activity overall than worms in pairs [6] might mean that we would expect to see *some* BrdU-labelled cells sooner in the octets than in the pairs (i.e., we are sampling from a bigger pool of elongating cells). This, however, would require that different cohorts of cells within the testis divide and differentiate at different rates; in practice such a bias is very unlikely to have affected our results, because the onset of elongation occurs as a coordinated wave throughout the testis, and the switchpoint from (all) round to (all) elongating spermatids is a clear and easily-scored criterion irrespective of treatment group (see Fig. 1). For a small number of ambiguous cases (usually owing to poor labelling quality), both researchers re-examined the slides to arrive at a consensus elongation score. In five cases ambiguities about the elongation state could not be resolved, and these worms were excluded from subsequent analyses, yielding a final sample size of 118 worms that were approximately evenly distributed across social group size and chase time treatments (*n*_5,P_ = 20, *n*_5,O_ = 20, *n*_6,P_ = 17, *n*_6,O_ = 21, *n*_7,P_ = 20, *n*_7,O_ = 20).

### Statistical analysis

Differences between treatments in log-transformed testis area were assessed using an independent samples *t* test. Because the octet worms were also somewhat larger overall than paired worms (*t-*test assuming unequal variances: *t*_107.2_ = 2.73, *P* = 0.007), as has sometimes previously been observed in similar experiments (e.g. [34, 57]), we also tested for a difference between treatments in relative testis size, i.e. using residuals from a regression of the log-transformed testis area on log-transformed body area. A difference in the onset of spermatid elongation at 5d post-BrdU administration was assessed with a Pearson chi square test, and the effects of chase time and social group size treatment as well as their interaction on spermatid elongation status using a binomial generalized linear model (GLM). Note that a generalized linear mixed model (GLMM; with plate ID fitted as a random effect) assuming a binomial error distribution and logit link function, to control for potential plate effects on elongation status, produced qualitatively identical results to those reported for the GLM and is therefore not shown. Similarly, in the analysis presented for day 5 worms only, the octet worms exhibiting elongated spermatids were distributed across all six plates, so again we can exclude that any strong ‘plate effects’ contributed to the pattern observed. All statistical analyses were performed in JMP (version 11) or, for the analysis of the spermatogenesis assay, using the glm and lme4 packages in R (version 3.1.3).

### Availability of supporting data

The datasets supporting the results of this article are available as Additional files 1 and 2.

## Additional files

Additional file 1: Morphological data on body and testis area. (XLSX 51 kb) Additional file 2: Immunocytochemical data on presence of elongated spermatids. (XLSX 41 kb)
